# A COMPARATIVE STUDY BETWEEN SANTULLI ILEOSTOMY AND LOOP ILEOSTOMY IN NEONATES WITH MECONIUM ILEUS

**DOI:** 10.1590/0102-672020200003e1538

**Published:** 2020-12-18

**Authors:** Shahnam ASKARPOUR, Amin AYATIPOUR, Mehran PEYVASTEH, Hazhir JAVAHERIZADEH

**Affiliations:** 1 Pediatric Surgery; 2 Pediatric Gastroenterology and Alimentary Tract Research Center, Ahvaz Jundishapur University of Medical Sciences, Ahvaz, Khouzestan, Iran

**Keywords:** Ileostomy, Meconium ileus, Cystic fibrosis, Infant, newborn, Ileostomia, Íleo meconial, Fibrose cística, Recém-nascido

## Abstract

**Background:**

Meconium ileus is a common cause of intestinal obstruction in neonates that different surgical methods have been described for its management such as Santulli and loop ileostomy.

**Aim:**

To evaluate and compare clinical efficacy of Santulli and loop ileostomy in neonates with meconium ileus.

**Methods:**

In this retrospective study, 58 patients with meconium ileus were evaluated. After analyses of hospital records, 53 patients with completed hospital records were included. Demographic information, surgery parameters and postoperative complications were extracted from the hospital records or calling parents.

**Results:**

Skin excoriation (21.4% vs. 84%, p<0.001), ostomy prolapsed (0 vs. 28%, p=0.003), and surgical site infection (7.1% vs. 28%, p=0.044) was significantly lower in Santulli ileostomy group. Furthermore, ileostomy output in first week (70.53±15.11 ml vs. 144.6±19.99 ml, p<0.001) and in 4^th^ week (2.14±4.98 ml vs. 18.4±17.95 ml, p<0.001) was significantly lower in Santulli ileostomy group as compared to loop ileostomy group. Finally, hospital stay in Santulli ileostomy group was 12±2.34 and in loop ileostomy 14.24±1.47 days (p<0.001).

**Conclusion:**

Santulli ileostomy is better than loop ileostomy due to significant less frequency of surgical site infection, skin excoriation, prolapse of ostomy, ileostomy volume output and hospitalization time.

## RESUMO

**Racional:**

O íleo meconial é causa comum de obstrução intestinal em neonatos e diferentes métodos cirúrgicos foram descritos para seu manejo, como Santulli e ileostomia em alça.

**Objetivo:**

Avaliar e comparar a eficácia clínica de Santulli e ileostomia em alça em neonatos com íleo meconial.

**Métodos:**

Neste estudo retrospectivo, foram avaliados 58 pacientes. Após análise, 53 pacientes com prontuários hospitalares completos foram incluídos. Informações demográficas, parâmetros cirúrgicos e complicações pós-operatórias foram extraídos dos prontuários ou dos pais por telefone.

**Resultados:**

Escoriações cutâneas (21,4% vs. 84%, p<0,001), estomia prolongada (0 vs. 28%, p=0,003) e infecção do sítio cirúrgico (7,1% vs. 28%, p=0,044) foram significativamente menores no grupo ileostomia Santulli. Além disso, a produção de ileostomia na primeira semana (70,53±15,11 ml vs. 144,6±19,99 ml, p <0,001) e na quarta semana (2,14±4,98 ml vs. 18,4±17,95 ml, p<0,001) foi significativamente menor no grupo de ileostomia Santulli em comparação com o de ileostomia em alça. Finalmente, o tempo de internação no grupo de ileostomia de Santulli foi de 12±2,34 e na ileostomia de alça de 14,24±1,47 dias (p<0,001).

**Conclusão:**

A ileostomia de Santulli é melhor que a em alça, devido à menor frequência significativa de infecção do local cirúrgico, escoriação cutânea, prolapso da ostomia, volume da ileostomia e tempo de internação.

## INTRODUCTION

Meconium ileus is a cause of intestinal obstruction in neonates due to cystic fibrosis and other factors that causes intraluminal accumulation desiccated meconium^4^ ^,^ ^13^. Different complications have been described for meconium ileus such as volvulus, atresia, perforation and meconium cyst. The first therapeutic option for uncomplicated meconium ileus is gastrografin enema which causes important side effects such as necrotizing enterocolitis, perforation, shock and occasional death^1^ ^,^ ^8^. Another option for management of uncomplicated status is surgical methods, including Bishop-Koop and loop ileostomy, Santulli or Mikulicz procedure. These are extensive operations associated with resection of dilated ileum and reduction of length of the gut and high stoma output. Moreover, in cases with meconium ileus, surgeons are faced with difficult decision to perform stoma or not, which causes worse complications in some cases including stoma retraction, prolapse, or necrosis^2^. Furthermore, a second surgery to close the stoma is required in some surgical procedures such as Bishop-Koop ileostomy, which increase some other complications such as fever, wound infection, leak from ileostomy closure, intestinal obstruction, and death^9^ ^,^ ^10^. On the other hand, some other surgical procedures such as Santulli ileostomy do not require gut resection and there is no intraperitoneal anastomosis and second operation^5^. Therefore, the rate of complications and outcomes differ among surgical procedures that comparing different procedures in the terms of outcomes, lead us to choose the best method with lower complications. 

As to best of our knowledge, there isn’t enough prospective study about the efficacy of two surgical methods, such as loop ileostomy and Santulli procedure, which are reported as the best operation options. Therefore, this study was designed to evaluate and compare clinical efficacy of Santulli ileostomy and loop ileostomy in neonates with meconium ileus.

## METHOD

The study received ethics approval from the Ethics Committee of Ahvaz Jundishapur University of Medical Sciences (IR.AJUMS. REC. 1396.1071)

### Study design and target group

This is retrospective analysis conducted in Surgery Department of Ahvaz Imam Khomeini Hospital, South-East of Iran from November 2014 to May 2017. The surgical outcomes of neonates with meconium ileus underwent Santulli ileostomy were compared to cases submitted to loop ileostomy. 

Inclusion criteria consisted of neonates with diagnosis of meconium ileus operated thought one of these ileostomies, Santulli or loop, and had completed hospital records. Exclusion criteria consisted of parental dissatisfaction to participate in the study, preterm cases, low birth weight neonates, other congenital anomalies, cases with defective hospital records, cases lost to follow-up, or with uncompleted data.

### Participants

**FIGURE 1:**
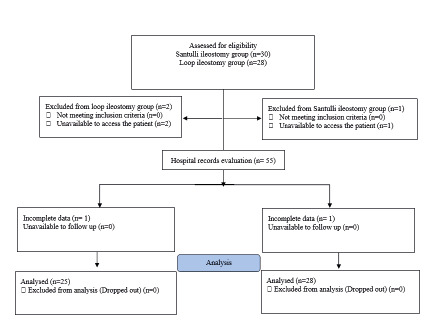
Study flowchart

The study flowchart is shown in Figure 1. Fifty eight patient’s hospital records with a diagnosis of meconium ileus, who had been diagnosed by pediatric surgeon based on clinical and paraclinical findings and entered in the inclusion and exclusion criteria, were included. Then, based on the surgical description, hospital records were divided into two surgical groups: Santulli or loop ileostomy 

After evaluating hospital records, 53 cases with completed hospital records (after calling parents in cases with incomplete data) were included, being 28 in Santulli ileostomy group and 23 in loop ileostomy group. 

Demographic information, postoperative complications, such as skin excoriation, ostomy prolapsed, stoma retraction, necrosis, surgical site infection, anastomotic leak, ileostomy output, duration of hospitalization, and adhesive intestinal obstruction, were evaluated in both groups.

In performing Santulli ileostomy, the proximal loop of divided bowel is brought out as ostomy and the distal end re-anastomosed to proximal bowel from 5 cm proximal to the ostomy site. In performing loop ileostomy, both bowel loops were exteriorized as ostomies. 

### Statistical analysis

Data were analyzed and reported only for patients with completed information. Statistical analysis of data was performed using SPSS version 22 software (SPSS Inc., Chicago, IL, USA). Chi-square test was used to compare qualitative variables between groups. Kolmogorov-Smirnov test was used in order to evaluate the normal distribution of all quantitative studied parameters. Student t-test was used for variables with normal distribution; on the other hand Mann-Whitney and Wilcoxon tests were used for variables without normal distribution. p-value less than 0.05 was considered significant.

## RESULTS

Demographic features in terms of age (p=0.899) and gender (p=0.833) both groups were similar (Table 1). Fifty five patients were dropped out and finally, 53 completed the study. Results showed that complications such as necrosis, anastomotic leak, adhesive intestinal obstruction (p>0.05) did not differ between the groups. But, skin excoriation (21.4% vs. 84%, p<0.001), ostomy prolapsed (0 vs. 28%, p=0.003), and surgical site infection (7.1% vs. 28%, p=0.044) was significantly lower in Santulli ileostomy group as compared to loop group. Furthermore, ileostomy output in first week (70.53±15.11 ml vs. 144.6±19.99 ml, p<0.001) and in 4^th^ week (2.14±4.98 ml vs. 18.4±17.95 ml, p<0.001) was significantly lower in Santulli ileostomy group as compared to loop group. Finally, hospitalization in Santulli ileostomy group was 12±2.34 days and in loop ileostomy was 14.24±1.47 day (p<0.001).

**TABLE 1:** Studied variables during different periods of time in both Santulli and loop ileostomy groups

Groups Variables		Santulli ileostomy (n=28)	Loop ileostomy (n=25)	p
Age (day)		8.57 ± 5.24	8.24 ±4.81	0.899
Gender (male)		16 (57.1%)	15 (60%)	0.833
Skin excoriation		6 (21.4%)	21 (84%)	<0.001
Ostomy prolapsed		0	7 (28%)	0.003
Necrosis		0	3 (12%)	0.098
Surgical site infection		2 (7.1%)	7 (28%)	0.044
Anastomotic leak		0	3 (12%)	0.098
Ileostomy output (ml)	1st week	70.53±15.11	144.6±19.99	<0.001
	4th week	2.14±4.98	18.4±17.95	<0.001
Adhesive intestinal obstruction		0	2 (8%)	0.089
Hospitalization (day)		12±2.34	14.24±1.47	<0.001

## DISCUSSION

According to our results, Santulli ileostomy had better effects on ileostomy output as compared to loop ileostomy method. Moreover, the rate of surgical complications and hospitalization were significantly lower in Santulli ileostomy as compared to loop ileostomy.

In the study performed by Kumar et al.^6^ reported that Santulli ileostomy is effective with lower morbidity and complication rates especially skin excoriation, ostomy prolapsed, and failure to thrive as compared to loop ileostomy, which can be used as an alternative to loop ileostomy. In another work performed by Eltayeb et al ^3^. showed that using Bishop-koop and Santulli surgery methods improve the survival of neonates with intestinal atresia and minimized the postoperative complications such as intestinal leak, delayed intestinal transit, septicemia, and disseminated intravscular coagulopathy. Rygl et al.^12^ showed that Santulli ileostomy is an effective and safe surgery technique for treatment of extremely low birth weight neonates with intestinal perforation. In the term of describing best management of uncomplicated meconium ileus, Hasan et al.^5^ reported that mean operation time, and mean time to start bowel movements were significantly lower in Santulli surgery methods as compared to Bishop Koop ileostomy, while postoperative complications, times to establish oral feeding, and irrigation tube removal showed no significant difference. Finally they concluded that Santulli ileostomy is effective and safe procedure for the management of uncomplicated meconium ileus. Mak et al.^7^ showed that in neonates with uncomplicated meconium ileus unrelieved by contrast enema, the Santulli ileostomy is an effective and safe treatment. Rondelli et al.^11^ demonstrated that conventional loop ileostomy increases the time required for the emission of gases and faeces through the stoma, and clinical anastomotic leakage, and postoperative complications. Vijayraj Patil et al.^9^ reported that tube ileostomy is effective and feasible as a diversion procedure as compared to classical loop ileostomy and has reduced morbidity. All these results are similar to our results. We found that Santulli ileostomy gives best cosmetic results with minimal complications and better than loop ileostomy method for management uncomplicated meconium ileus.

While, in an article published by van de Pavoordt et al. ^14^ showed that overall complication rate after loop ileostomy was 17% with 13% early postoperative complications, moreover the major complication was obstruction and abdominal septic complications. They concluded that loop ileostomy closure is a safe operation with a low morbidity. However, we found higher complication rates in loop ileostomy closure, which may due to different sample size, different demographic features, and controlling confounding variables. 

Study limitation was being done in a single center with low family compliance and long term complications not included. Therefore, another prospective clinical trial with higher sample size is needed in order to control confounding variables and dividing patients into two groups based on random method.

## CONCLUSION

There are beneficial effects of Santulli ileostomy technique over loop ileostomy in postoperative outcomes, best cosmetic results with minimal complications. In short term, significantly it leads to better control of surgical complications, and in longer follow up increases the quality of life. 
